# Cation–π interactions drive hydrophobic self-assembly and aggregation of niclosamide in water†

**DOI:** 10.1039/d1ra05358b

**Published:** 2021-10-07

**Authors:** Said A. H. Vuai, Mtabazi G. Sahini, Isaac Onoka, Lucy W. Kiruri, Daniel M. Shadrack

**Affiliations:** Department of Chemistry, College of Natural and Mathematical Sciences, University of Dodoma P. O. Box 338 Dodoma Tanzania; Department of Chemistry, Kenyatta University P. O. Box 43844-00100 Nairobi Kenya; Department of Chemistry, Faculty of Natural and Applied Sciences, St. John’s University of Tanzania P. O. Box 47 Dodoma Tanzania dmshadrack@gmail.com mshadrack@sjut.ac.tz

## Abstract

The beneficial medicinal effects of niclosamide have been reported to be hampered by poor aqueous solubility and so a higher concentration dosage is required. In this work, we have studied the aggregation properties of niclosamide in water by varying the number of monomers. We have employed all-atom classical molecular dynamics simulation in order to explore such properties. The equilibrium structure exists in an aggregated state with structural rearrangements of the stacking units. Niclosamide monomers tend to form clusters in an orderly manner and tend to aggregate in parallel and antiparallel orientations of the phenyl rings as the monomers are increased in number from 4 to 9. Upon increasing the size from 9 to 14, and from 49 to 150, a considerable dominance of the metastable parallel arrangement is observed, resulting in the formation of a closely packed cluster with hydrophobic contacts. The metastable conformation self-arranges to a T-shape before forming a stable planar antiparallel displaced conformation. The aggregated π–π parallel and cation–π antiparallel clusters in water exist in a β-conformer. We further observed that formation of a stable cluster aggregate entails the formation of an intermediate metastable cluster that disperses in solution forming a large stable cluster. We also discovered that movement of the water is faster in less aggregated clusters and as the cluster size increases, the mobility rate becomes much slower.

## Abstract 2

In this work, we have studied the aggregation properties of niclosamide in water by varying the number of monomers.

## Graphical abstract

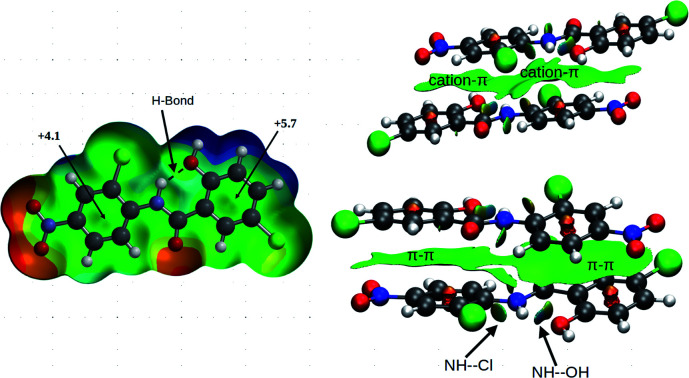

## 1 Introduction

**Fig. 1:**
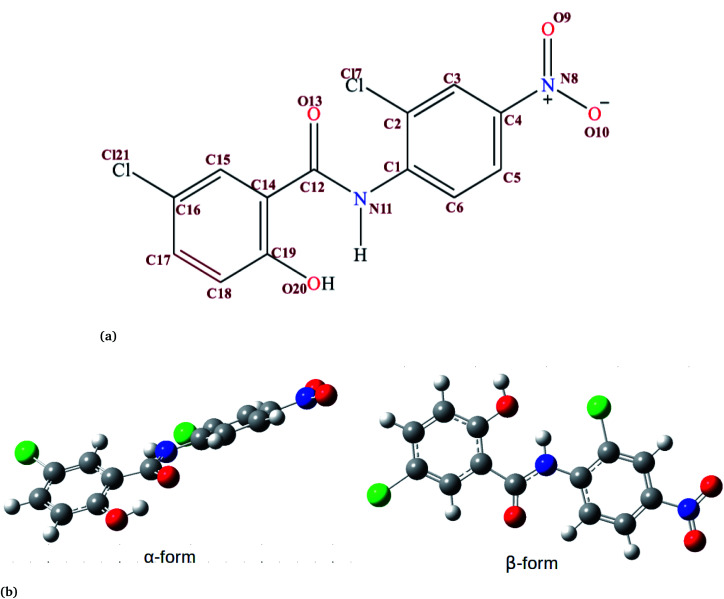
(a) The chemical structure of niclosamide. (b) Crystal structure of two forms of niclosamide. The β-conformer is formed when the hydroxyl group (O20) from the ring forms a hydrogen bond with the hydrogen from a nitrogen (N11) at the middle, while the α conformer is formed when the hydroxyl group (O20) forms a hydrogen bond with the keto group (C12=O13). Atoms are shown by color: oxygen in red, nitrogen in blue, chlorine in green, carbon in grey and hydrogen in white.

**Fig. 2:**
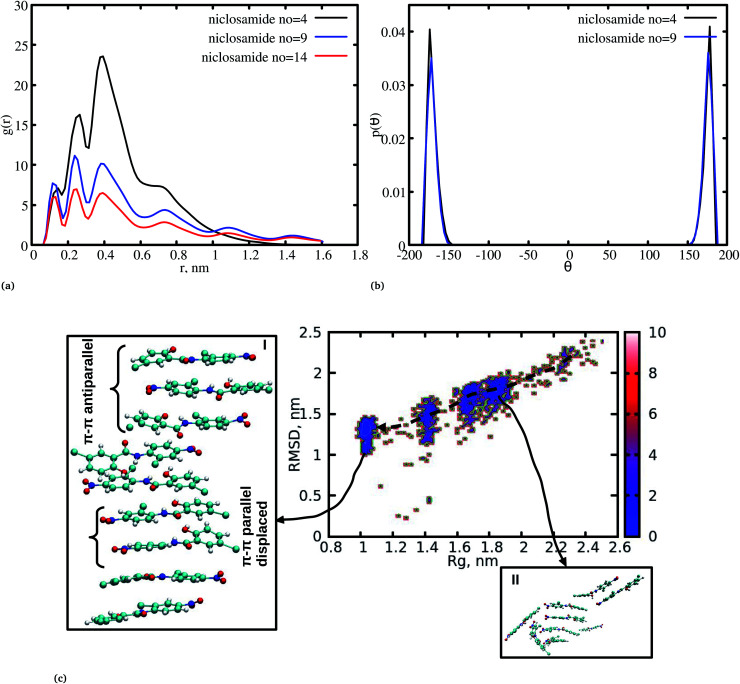
(a) Radial distribution function (RDF) for niclosamide systems (4 to 14), (b) dihedral angle distribution for niclosamide systems, the system formed by 14 niclosamide monomers is omitted for clarity. (c) Multi-step self-assembly process of niclosamide in water forming an aggregated state.

Niclosamide (NC) (Fig. 1, 2,5-dichloro-4-nitrosalicylanilide) is a drug that the food and drug authority (FDA) has approved as an anthelminthic.^1,2^ NC is included in the WHO’s list of essential medicines. It has so far been considered as one of the most highly potent multifunctional drugs.^3^ NC belongs to the Biopharmaceutical Classification System (BCS) Class II, meaning that it has a low solubility and high permeability.^4^ The crystalline form in which most drugs exist affects their solid-state characteristics.^5,6^ Generally, the way drugs react plays a significant role on their performance characteristics. NC has been reported to crystallize into two solvate forms: two monohydrates (H_A_ and H_B_) and one anhydrous.^7^ In the monohydrate form of NC, the water molecule is bound at the asymmetric unit of the drug surroundings by van der Waals envelopes of other drug molecules. The existence of NC in these different forms affects the dissolution characteristics and its bioavailability.^6,8^

In addition to its anthelminthic property, NC has been reported to be useful in the treatment of various categories of cancer and bacterial, fungal, and viral infections.^9–11^ The efficacy of NC in the treatment of viral infections has been discussed in more detail, particularly in the treatment of coronavirus disease 19 (COVID-19) caused by SARS-CoV-2. The anti-viral properties of NC against SARS-CoV has been reported by Xu *et al.*^12^ and Wu *et al.*,^10^ with an IC_50_ of 1.56 μM. Also, NC was reported to inhibit the replication of MERS-CoV.^13^ Building from the existing knowledge about its potential anti-viral properties, NC was investigated for potential anti-viral activity against SARS-CoV-2.^14,15^ Ko *et al.*, reported the anti-SARS-CoV-2 activity of NC with an IC_50_ of 0.28 μM.^14^ A recent study by Pindiprolu *et al.*^16^ has suggested possible mechanisms for the anti-SARS-CoV-2 activity of NC as blocking the endocytosis of SARS-CoV or preventing its autophagy through inhibition of the S-phase kinase-associated protein 2 (SKP2).^16^ The suggested mechanisms were developed from the analogy of MERS-CoV and SARS-CoV. Even though NC is a potent multifunctional drug, it is practically insoluble in water but slightly soluble in diethyl ether, ethanol, chloroform, tetrahydrofuran (THF) and dioxane.^6^ It has been reported that NC exhibits water solubilities of about 13.32 μg mL^−1^ and 0.6–0.9 μg mL^−1^ for the anhydrous and monohydrate forms, respectively.^17^ Due to its poor water solubility property the oral absorption of NC is limited. NC is mainly prepared in two types of dosage form: tablets and suspensions.^7^ The drug formulation of NC involves use of either NC anhydrate or the NC monohydrate forms.^18^ Due to the water affinity characteristic of the anhydrous crystal form of NC, the suspension develops into a cement-like sediment during formulation or when stored.^17^ Such limitations can be overcome by introducing an organic solvent, encapsulating with organic materials or coating with water-insoluble organic molecules. Various approaches have been applied to increase the solubility and hence the bioavailability of NC. They include: the use of lipid emulsions,^19^ solid–lipid nanoparticles,^4^ micelles,^20^ nanoparticles,^21^ nanosuspensions,^22^ nanocrystals^23^ and co-crystals.^18,24,25^ However, little is known about how NC aggregates in an aqueous environment.

The aggregation process of NC needs to be addressed by atomistic simulation approaches which provide a better understanding of the characteristics of various materials at a molecular level. Computational investigation of biomolecular aggregation provides better microscopic details than the experimental approach. Problems related to aggregation and solubility have also been previously addressed using computational methods^26^ for other systems. In order to address the problem of niclosamide aggregation at the atomistic level, we have carried out a molecular dynamics simulation of niclosamide monomers in water at an NPT ensemble for 50 ns.

## 2 Computational methods

### 2.1 Molecular dynamics simulation

In this study, molecular dynamics simulation was used to provide atomistic information on the hydrophobic interactions of niclosamide aggregation as well as the hydrophobic hydration that supports the stability. The structure of the niclosamide monomer was obtained from the pubChem database^27^ in sdf format, energy minimized and converted to pdb file format. The coordinates and topology of niclosamide were obtained using the antechamber tool^28^ together with the general AMBER force field (GAFF),^29^ using the AM1-BCC charge method.^30^ The obtained topology and coordinates were converted to Gromacs file format using the AMB2GMX python script. All the simulations reported were carried out at a temperature of 300 K and 1 bar pressure. Three individual systems comprising 4, 9 and 14 monomers of niclosamide were separately solvated in a cubic box containing a TIP4P water model.^31^ The box size was 3.0 × 3.1 × 3.0 nm for 4 monomers, 3.2 × 3.2 × 3.2 nm for 9 monomers, 3.5 × 3.5 × 3.5 nm for 14 monomers, 4.27 × 4.27 × 4.27 nm for 49 monomers and 10 × 10 × 10 nm for 150 monomers. The systems were energy minimized using a steepest descent method, and were equilibrated at an NPT ensemble for 500 ps. The temperature and pressure coupling were held using the v-rescale thermostat and Parinello–Rahman barostat, respectively.^32,33^ A production run was carried out using the NPT ensemble for 50 ns with an integration time step of 2 fs. Periodic boundary conditions (PBCs) were applied in all directions. The Particle Mesh Ewald (PME) method was used to treat long-range electrostatic interactions with a cutoff distance of 11 Å for both electrostatic and van der Waals interactions, while covalent bonds were constrained using the LINCS algorithm.^34,35^

The 2D free energy landscape was computed using the following equation;

1 *F* = −*k*_B_*T* ln *P*_(r)_

where, *k*_B_ is the Boltzmann constant, *T* is the temperature and *P*_(r)_ is the probability distribution of the distances measured.

### 2.2 Quantum mechanical calculations

Geometry optimizations of the α and β-form were calculated using the Density Functional Theory (DFT) method at the M06-2X/def2-TZVP level of theory using the Gaussian 16 series of programs.^36^ The Grimme’s dispersion correction^37^ was also employed using the keyword empirical dispersion = gd3 in order to evaluate noncovalent interactions dominated by dispersion effects such as π-stacking. The interaction energies were corrected for the basis set superposition error (BSSE) using the counterpoise correction.^38^ All the calculations were evaluated in an implicit water environment using the polarizable continuum model (PCM).^39^ To account for solubility, the Gibbs free energy of solvation was computed at M062X/6-31G* using the implicit solvent (keyword “SCRF = SMD”).^40^

## 3 Results and discussion

### 3.1 Hydrophobicity, niclosamide–niclosamide interaction and orientation

The radial distribution function (RDF) is an important tool for analysing the molecular interaction of niclosamide. Herein, the RDF was calculated by counting the number of atom pairs in niclosamide or niclosamide–water solvents at a specific distance. The density probability of atoms was then averaged as a function of distance. Fig. 2a shows the distribution of niclosamide molecules. It can be observed that the hydrophobic core of niclosamide with monomers arranged in parallel and antiparallel π–π stacking continuously reduces as the number of monomers increases from 4 to 14. The figure also shows three prominent peaks at three different positions suggesting the formation of three layers of clusters distributed in the system. Interestingly, we observed that, within the same system, the probability density peaks have different heights. This is attributed to the fact that as the cluster grows, it becomes more compact, and the monomers become closer to each other. However, as the system size increases from 9 to 14, the peak heights remain relatively similar suggesting that the system is closely packed (Fig. 2a).

Upon comparing the three systems, one can observe that as the system size increases, the intensity of the RDF peak decreases. On the other hand, a high intensity RDF peak relates to the loose system with NC = 4, a similar observation has also been reported for a loose system of germanium atomic structures where the intensity of the RDF peak was also high.^41^

In order to understand how individual monomers adhere to each other in the aggregate, we calculated the dihedral angle distribution (C14, C12, N11 and C1, see Fig. 1a) that connects the two phenyl rings. Fig. 2b shows a bimodal distribution suggesting structural alignment of the two phenyl rings to each other. Visual inspection of the snapshot showed two types of phenyl–phenyl orientation existing in π–π antiparallel stacking and π–π parallel-displaced stacking (Fig. 2c). It is also important to highlight that, as the system increased from 4 to 9 the formation of parallel and antiparallel π–π stacking was equally observed from the equilibrium system (Fig. 2c). It is worth noting that, as the system size increased from 9 to 14, the metastable parallel conformation dominated, followed by the T-shaped conformation, which occurred for a short time before transforming to the stable anti-parallel conformation (Fig. S1†). The observed T-shape conformation clearly resembles those observed in proteins.^42^ The formation of this aggregate is facilitated by strong hydrophobic π–π interactions among the phenyl rings of niclosamide, providing increased stability.

**Fig. 3:**
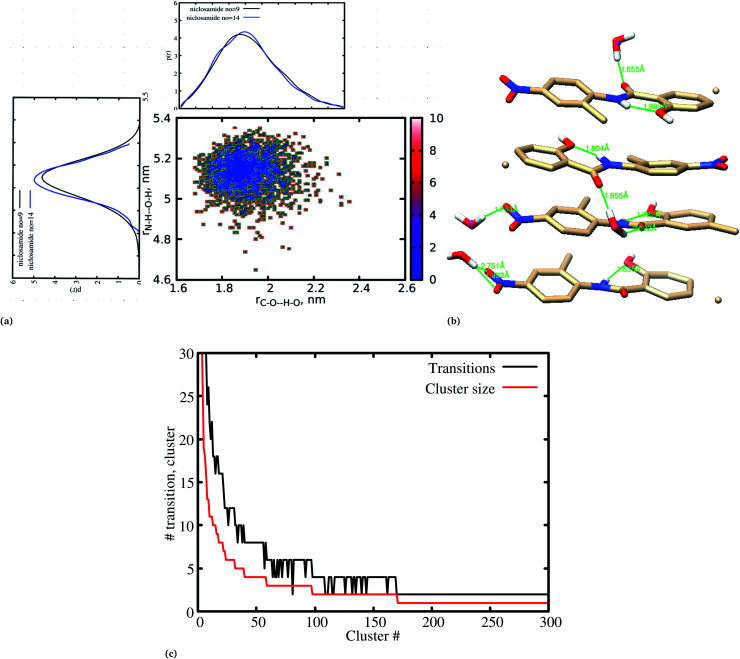
(a) A 2D free energy landscape showing the preferred conformation for niclosamide in water, (b) the intramolecular and intermolecular hydrogen bonds formed between the hydroxyl group and the nitrogen group of niclosamide as well as the keto group with water molecules. (c) Cluster size and transition formed during the aggregation process of niclosamide.

Previous experimental studies on the crystal structure of niclosamide have reported two different results on conformational stability. Kosheleva *et al.*^43^ crystalized niclosamide and observed two conformations, the α and β-conformation, and noted that the latter conformation was thermolabile and easily transformed to the stable α-conformation. Later on, Caira *et al.*^8^ crystalized niclosamide in different solvent and only observed the β-conformation. Our analysis based on the aggregated self-assembly behavior suggests the existence of the β-conformation only (Fig. 3a and b), which is in good agreement with experimental report by Caira *et al.*,^8^ who concluded that “the preferred conformation of the niclosamide molecule in the pseudopolymorphs investigated is the β-form”. It is worth noting that the pseudopolymorph packing of niclosamide in a different solvent reported by Caira *et al.*,^8^ is in good agreement with the packing aggregates reported in our work, and to some extent shows a similar packing motif to that observed in our simulation. The keto group formed hydrogen bonds with water molecules as seen in Fig. 3b, therefore stabilizing the observed π–π stacking, hence favouring the formation of the β-conformation only. These observations are similar to the experimental crystal structure of niclosamide in water, whereby the keto oxygen was observed to form a hydrogen bond with water.^8^ Although, in this study we have explored the self-assembly of niclosamide in water and extending and exploring the crystal structure stability at the atomistic level is warranted. This will further provide a better insight on the stability of the two forms of niclosamide in water.

### 3.2 Cluster growth from segregation to stable aggregation

Cluster aggregation of several organic molecules such as caffeine has been studied using experimental methods. However, the transition process from segregation to aggregation is inadequately addressed from an experimental point of view. In this work, we have monitored the process of niclosamide aggregation using atomistic simulation.

**Fig. 4:**
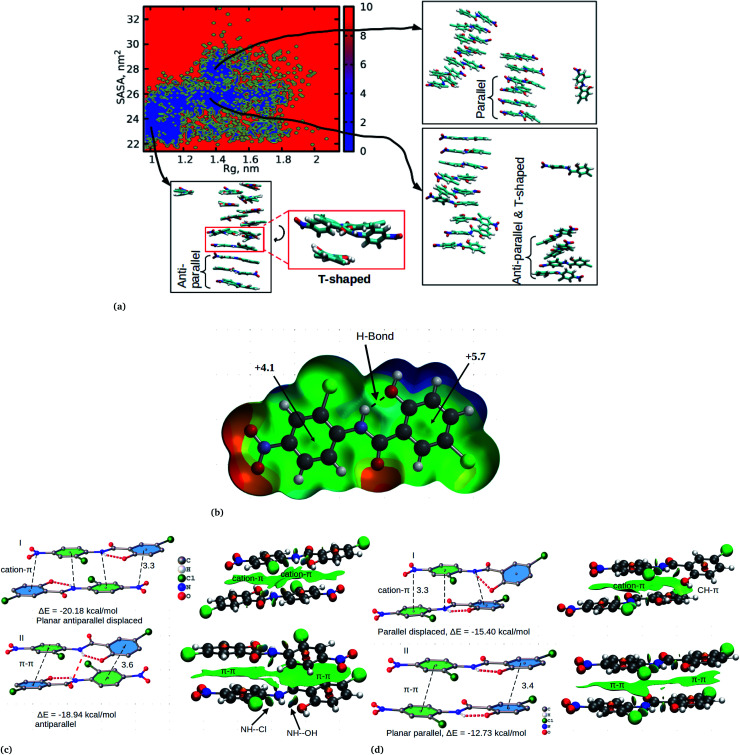
(a) The formation of a stable aggregated system of niclosamide with 14 monomers. The aggregation is a multi-step process whereby individual monomers bind to form small aggregates dominated by the formation of parallel and T-shape conformations. With time, the small aggregates join to form a large cluster and re-orient to build a stable planar anti-parallel displaced conformation. (b) MEP surface of niclosamide, the energies shown at different points are in kcal mol^−1^. (c and d) Interactions and arrangements of niclosamide dimers, the distance shown is in Å. To the right side of each figure is the NCI plot showing the interactions.

We have also analyzed the cluster size and transition from one cluster to another to provide a better understanding. We observed a correlated dynamic behavior between cluster size and transitions; as the number of clusters decreased (from monomer to aggregated structure), the number of transitions also decreased, until a single cluster was formed and no further cluster growth was observed (Fig. 3c). The aggregation process of niclosamide resembles the nucleation process of a liquid, whereby each monomer tends to aggregate and segregate in the course of time until it attains an optimal cluster size.^26^ A similar observation for curcumin resembling nucleation growth of liquid was previously studied using atomistic simulation.^26^ The formation of stable aggregated niclosamide from the monomers was monitored by calculating the free energy landscape as a function of radius of gyration (*R*_g_) and the root mean square deviation (RMSD) and solvent accessible surface area (SASA), which measures the degree of aggregation/segregation. Fig. 2c shows that the most aggregated system with 9 niclosamide monomers corresponds to lower *R*_g_ and RMSD values. However, this is a multi-step process involving the formation of three metastable steps before reaching the final cluster. The structures with larger *R*_g_ values are more segregated, however, with time, monomers assembled themselves until a final cluster was formed. The metastable states (Fig. 2c) showed that the monomers tended to join a larger stable state; while some monomers remained isolated for a longer time. Similarly, FES as the function of *R*_g_ and the SASA for the system with 14 niclosamide monomers shows that with large *R*_g_ values the system is in its segregated form. However, as the *R*_g_ values decreased the system attained larger stable aggregations. As observed from the 1D FES for the SASA (Fig. S2†), the most stable aggregate shows two minima with SASA values of 23 and 25 nm suggesting that the aggregated system might exist in two conformational states. Visual inspection of the aggregated form with SASA value = 25 nm (Fig. 4a) showed two conformational states, the parallel and anti-parallel, where the latter exists in a metastable state. The T-shaped conformation was also observed in the metastable state. Both parallel and T-shaped conformations were observed to be isolated and resulted in small aggregated clusters. We hypothesize that, after gaining some enthalpic and entropic stabilizations, the isolated monomers are able to bind to the large cluster and form a stable single aggregate. This is observed at a SASA value of 23 nm, where all monomers have joined, forming a stable anti-parallel conformation. In general, our MD calculations for 4, 9 and 14 monomers suggested that niclosamide forms a stable aggregated structure which tends to exist in an anti-parallel displaced form.

**Fig. 5:**
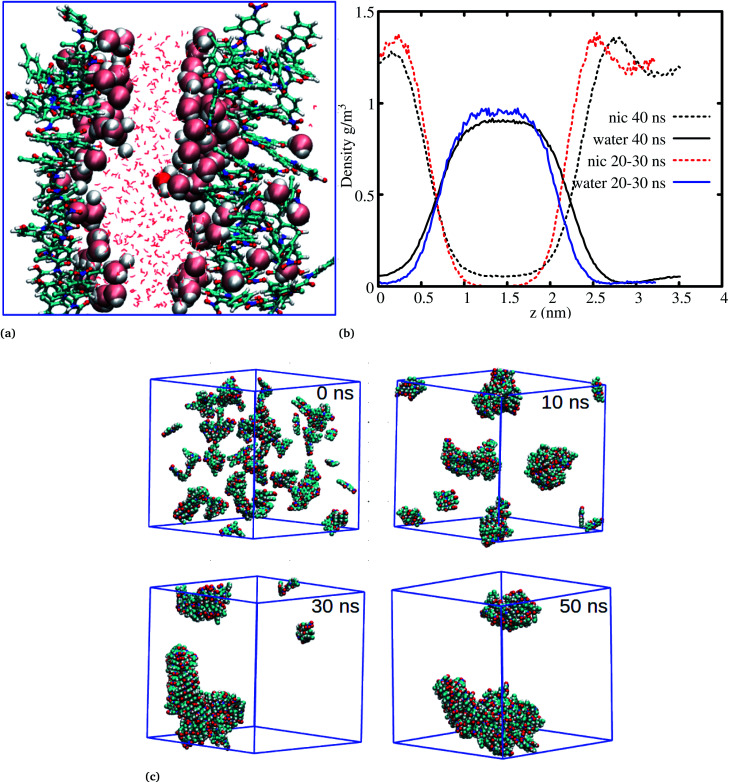
The aggregation behaviour of niclosamide in water. (a) The aggregation of 50 monomers of niclosamide, during the 60 ns niclosamide formed two clusters separated with water molecules. The two clusters are formed near the edge of the box leaving water molecules at the center. Bulk water molecules are also penetrating the large cluster to hydrate the other side. (b) Density profiles of niclosamide and water molecules at different times. (c) The aggregation process of 150 molecules of niclosamide, the process resembles the aggregation of 9 and 14 monomers, where small monomers joins to form a large cluster. During the 30 ns niclosamide monomers have formed a chair like cluster which continues to grow until 50 ns.

The systems discussed above helped to probe the interactions of the niclosamide molecule in water, however, in order to gain further insight into the aggregation behavior of niclosamide, large systems with 49 and 150 monomers of niclosamide were considered. Interestingly, as observed for systems with a small number of monomers, the larger system showed similar aggregation behavior and tendencies, however, with the formation of two well defined crystal like structures for the system with 49 monomers. Visual inspection showed that the aggregated systems are separated with bulk water, a phenomena which is not observed in small systems (Fig. 5a). To have a clear understanding of the interactions between niclosamide aggregates and water, density profiles were calculated along the *z* direction (Fig. 5b). Density profiles suggested that bulk water molecules tend to diffuse into the cluster surface of niclosamide over time. It is important to note from the density profile that, the two clusters formed are different sizes. As shown in the snapshot taken at 40 ns and in the density profile (Fig. 5a and b), it is evident that, the clusters formed do not have equal numbers of monomers, one cluster tends to have a larger number of well defined monomers with more penetrated hydrating water. The aggregation of 150 monomers further shows similar conformation (formation of antiparallel and parallel) arrangements with similar cluster growth observed compared to smaller systems, however, with a different nature. The larger system grows to form a chair like cluster, where small clusters join to form a larger thermodynamic cluster (Fig. 5).

In order to confirm the observed orientational interactions, quantum mechanical calculations based on DFT were performed on various conformations. First, in order to know the electrostatic nature of the π-system and the most electron rich/deficient regions of β-niclosamide, the molecular electrostatic potential (MEP) was computed (Fig. 4b). As observed for the MD calculations, the plotted MEP surface shows a hydrogen bond formed between the NH–OH groups. Interestingly, we noted that the six membered ring connected to the nitroso group (NO_2_) is less positive (+4.1 kcal mol^−1^) than the other six membered ring (+5.7 kcal mol^−1^) with the hydroxyl group (OH). This observation suggests that the formation of antiparallel cation–π or parallel π–π stacking displaced arrangements would be electrostatically favoured. As noted earlier, the antiparallel displaced dimer showed favourable interactions with the N atom of the nitroso group (which is less positive) which coincides with the location of the region of the more positive ring, forming a stable cation–π interaction. Eventually, this interaction is much favoured with a formation energy of −20.18 kcal mol^−1^ compared to its counterpart, the non-displaced π–π stacking interaction, which resulted in an energy of −18.94 kcal mol^−1^. However, the difference in energy is very minimal within the error bars. We further observed that the same conformation is likely to distort its planarity at the expense of forming an antiparallel hydrogen bond between the amide hydrogen and the carbonyl group of the other monomer. Such twisting and shifting reduces the previously observed cation–π interaction, which is the most stable, to the least stable π–π stacking interaction (Fig. 4c). A similar observation is noted for the parallel displaced conformation which is more stable (−15.40 kcal mol^−1^) compared to the parallel conformation (−12.73 kcal mol^−1^) (Fig. 4d). The observed antiparallel and parallel interactions were further characterized by computing the noncovalent interaction (NCI) plot index, which is an effective tool for showing noncovalent interactions. In Fig. 4c and d we show extended green isosurfaces located at the middle of the dimers characterized by acceptor and donor groups suggesting a large overlap of the π-interaction systems. It should be noted that the blue isosurfaces within the NCI plot represent formed intramolecular hydrogen bonds. Interestingly, our MD simulation is in reasonable agreement with the DFT calculation, where both suggest that the planar anti-parallel displaced conformation is the most stable. It should be noted that the observed T-shaped conformation is the metastable state changing from the parallel to anti-parallel conformation. Normally, the stable conformation for the niclosamide aggregate is stabilized by the formation of cation–π stacking interactions occurring in the displaced planar anti-parallel conformation. We have further observed that, on increasing the cluster size, the distances between planar fragments of parallel molecules decreased which indicates an indirect cooperativity in the hydrophobic contact (Fig. S4†). Experimental results on the crystal structure of the β-conformation showed that niclosamide exists in its planar conformation stabilized by the intramolecular hydrogen bond between the NH(11)–OH(20),^8^ which is in good agreement with both MD and DFT results.

### 3.3 Niclosamide cluster hydration

It has been noted that hydration has a great influence on cluster stability.^26,44^ We examined structural water near aggregated niclosamide to gain a better understanding of the effect of hydration on cluster stability. Since niclosamide contains hydrophilic groups that tend to point outward, the cluster surface is hydrophilic. The observed local water structures in the vicinity of hydrophilic and hydrophobic surfaces exhibit different characteristics.

**Fig. 6:**
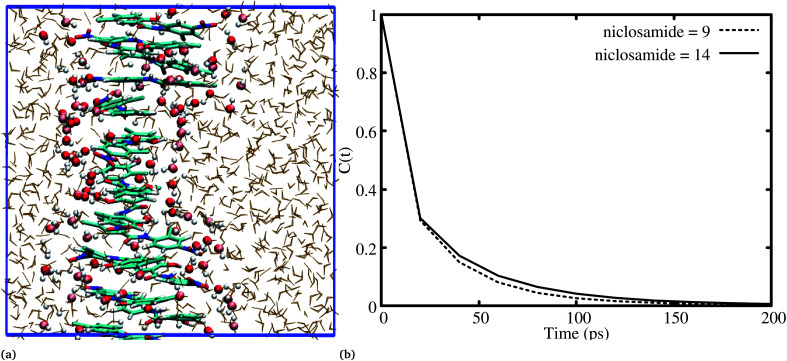
(a) Hydration of the niclosamide cluster with interfacial water, (b) a hydrogen bond autocorrelation function for niclosamide aggregated clusters with 9 and 14 monomers of niclosamide. Hydrogen bonds persist for all the systems until 20 ps, then there are faster dynamics for a system with 9 monomers as compared to 14 monomers.

Fig. 6a, shows that solvated water molecules tend to penetrate into different levels of the cluster. Although the penetration of water molecules is limited to the hydrophobic region, they penetrate through the hydrophilic nitroso region to form a network of hydrogen bonds with the keto oxygen and the nitroso group as shown in Fig. 3b. The resulting hydrogen bond networks play an important role in stabilizing the cluster. To provide further understanding of the hydrogen bond lifetime, the hydrogen bond autocorrelation function, *C*(*t*) for continuous hydrogen bonding was calculated using eqn (2) and then fitted to the second order Legendre polynomial function as previously reported by Rawat and Biswas.^45^ In eqn (2), *S*_*i*,*j*_(*t*) measures the existence of hydrogen bonds between the *i*th and *j*th atoms at time *t*, and it will be zero if hydrogen bonds are absent. In Fig. 6b, we demonstrate that the intermolecular hydrogen bond relaxes faster for a small cluster *i.e.* with 9 niclosamide monomers, however; as the cluster size increases, the amount of penetrated water also increases thereby forming dense hydrogen bonds and hence slowing the water movement. The hydrophilic groups formed relatively more stable hydrogen bonds as compared to the hydrophobic region.2
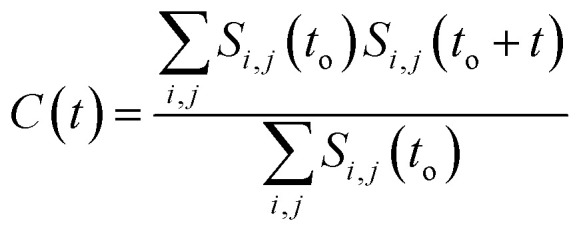


Fig. 8a, shows the water density near the cluster relative to the bulk solvent density. We observed that the cluster surfaces were hydrated. However, dewetting is observed to have taken place near the hydrophobic core. This is because hydrophobicity tends to break the already established hydrogen bond networks resulting in a decrease in density as the system size increases. It should also be noted that, although there is a wetting process, hydrophobic regions near the hydration site may affect the surrounding water density in the niclosamide cluster. We further observe that water in the hydration shell near the niclosamide cluster forms hydrogen bonds with the hydrophilic groups/sites. Fig. 8b shows the RDF between the niclosamide sites (C=O, O–H, –NH and –NO) and the water oxygen (OW). The only sites that form hydrogen bonds with the solvent are those belonging to the carboxylic and nitroso groups as seen in Fig. 3b. The carbonyl group shows a higher density of water indicating a strong hydrogen bond, followed by a hydroxyl group. The nitroso group (–NO) is found to have lower water density despite its exposure to the solvent. This is attributed to the reorientation of niclosamide groups. Furthermore, the –NH group was less hydrated and least exposed to the solvent surface as indicated in Fig. 8b.

### 3.4 Self-aggregation and solubility of niclosamide

**Fig. 7:**
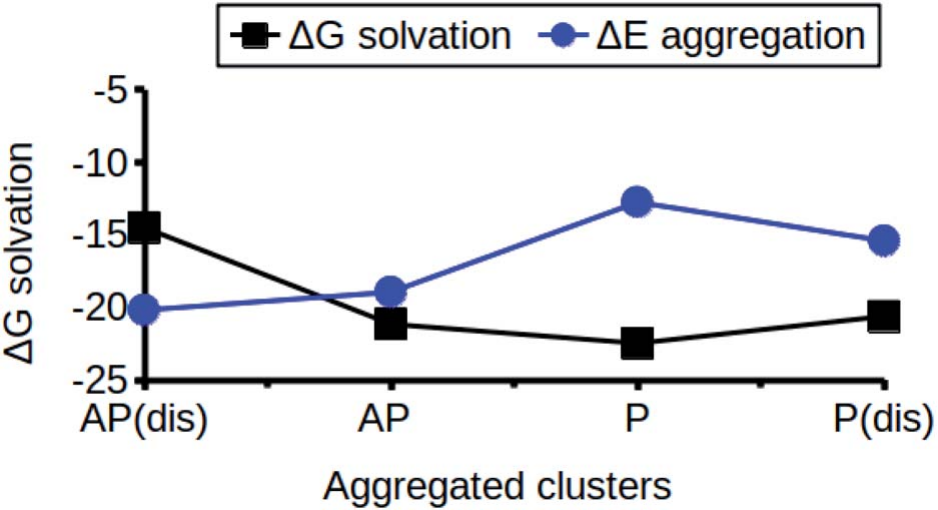
The relationship between niclosamide self-aggregation and solubility in water. Stable aggregates exhibited the lowest Gibbs free energy of solvation and *vice versa*. AP(dis) means antiparallel displaced, AP means antiparallel, P means parallel and P(dis) means parallel displaced.

The relationship between the self-association and the solubility issues of niclosamide in water is described. To gain insight into the solubility issues of aggregated niclosamide, we considered four different interaction modes observed in classical MD simulations of different cluster sizes, and calculated the Gibbs solvation free energy which relates to the solubility of niclosamide in water. The energy of interaction calculated from DFT for the four self-association modes; the antiparallel displaced (AP(dis)), antiparallel (AP), parallel (P) and parallel displaced (P(dis)) is shown in Fig. 4. As described above, the stable aggregate corresponds to the antiparallel displaced (−20.18 kcal mol^−1^) and antiparallel (−18.94 kcal mol^−1^) conformations. This suggests that the poor solubility of niclosamide in water is due to the antiparallel arrangements characterized by the formation of the cation–π and π–π interactions. To understand the relationship between the self-aggregation behavior and solubility issues, we have calculated the Gibbs free energy of solvation (Δ*G*_solv_). Interestingly, the more stable aggregated form of niclosamide, that is, the antiparallel displaced mode (AP(dis)) exhibited the lowest (*i.e.* more positive value of) Gibbs free energy of solvation (−14.48 kcal mol^−1^). On the other hand, the planar parallel aggregation mode showed increased solubility with a Gibbs solvation free energy of −22.47 kcal mol^−1^ (Fig. 7). As mentioned earlier, niclosamide clusters are hydrated, Fig. 3b shows that the parallel cluster is the most hydrated and interacted with water compared to the antiparallel conformation, which is in good agreement with the calculated Gibbs free energy of solvation for the parallel clustered conformation. We suggest that, the marginal solubility (13.32 μg mL^−1^) of niclosamide in water in its anhydrous form and 0.96 μg mL^−1^ in its monohydrated forms is associated with the planar parallel structure and the formation of π–π interactions. On the other hand, the poor solubility is due to the antiparallel arrangement which results in the formation of cation–π interactions. We hypothesize that the aqueous solubility of niclosamide could be increased by breaking the cation–π interactions and favouring the formation of planar parallel interactions, however, this problem needs to be explored in future.

### 3.5 Water dynamics at niclosamide clusters

The hydrogen bond autocorrelation function provided an insight into the dynamical behavior of interfacial and bulk solvents. To further understand the dynamical behavior of interfacial water near the cluster interface, we calculated the residence time of water at the interface in the first hydration shell and the bulk. The residence time of water was estimated by calculating the survival probability *N*_w_(*t*) as described by Rani *et al.*,^46^ The residence time correlation function *P*_*i*_(*t*_*n*_, *t*) is the conditional probability for each *i*th water molecule; if *P*_*i*_(*t*_*n*_, *t*) = 1 it indicates that the water remains within the hydration shell in the time interval *t*_*n*_ and *t*_*n*_ + *t*, otherwise it is zero.3
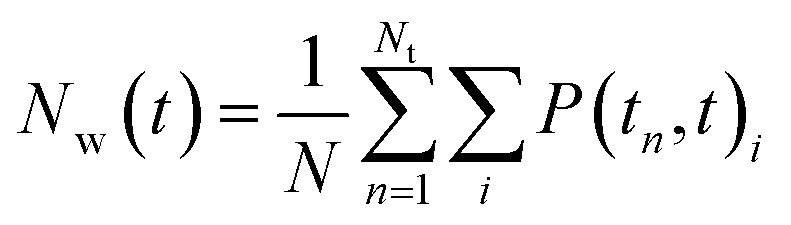


**Fig. 8:**
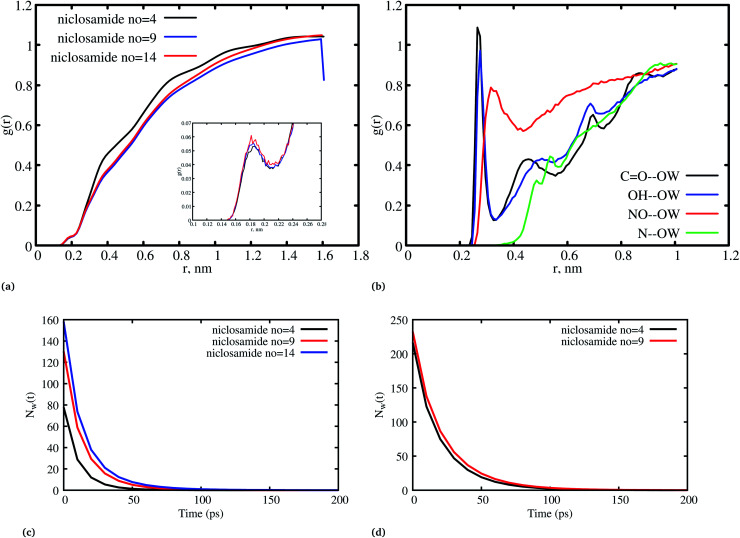
(a) The radial distribution function (RDF) between niclosamide and water oxygen (OW) for different systems, (b) the RDF between niclosamide sites (C=O, –OH, –NH, and –NO) and water oxygen (OW), (c) dynamics of the hydration water molecules that remained trapped within the first shell (3.5 Å), (d) dynamics of bulk water.

Fig. 8c and d shows the residence time correlation function of water at the cluster within the hydration shell and the bulk. The first hydration shell was defined for water within 3.5 Å and bulk water above 3.5 Å. Notably, different cluster sizes exhibited different dynamics of water. Although the hydrophobic tendency is pronounced, we observed that when niclosamide increased in size, the dynamics of water hydration reduced and remained intact for a longer time. At least 8 water molecules remained until 50 ps for the large cluster with 9 niclosamides. This observation is further supported by the calculated SASA values (Fig. S3†), where it is shown that the SASA values increase, while the cluster size increases. This implies that, an increase in size resulted in an increase of trapped water molecules which are responsible for hydrating the surface. This observation is similar to the hydrogen bond autocorrelation function. However, the dynamical behaviour of the bulk solvent did not show much difference for different clusters (Fig. 8c).

To gain insight into the timescale associated with the dynamics of water, the residence time correlation function was fitted to an exponential function as described previously by Rani *et al.*,^46^

4 *S*(*t*) = *a* exp(−(*t*/*τ*_s_)^*γ*^) + *b* exp(−(*t*/*τ*_2_)) + *c* exp(−(*t*/*τ*_3_)) + *n*_p_

where, *n*_p_ is the number of water molecules present at niclosamide throughout the simulation time, *τ*_s_ is the residence time for the decay of the stretched exponential. *τ*_2_ and *τ*_3_ are the residence times for slow biexponential decay for the first and second components, respectively. *γ* represents a quantitative measure of the deviation of the relaxation curve from a classical exponential function. The value of *γ* = 1 for a classical exponential function. A large *γ* value indicates the presence of system temporal disorders.^46^ The best fit parameters are presented in Table 1, the average residence time of the stretched exponential is obtained using the expression:^46^5
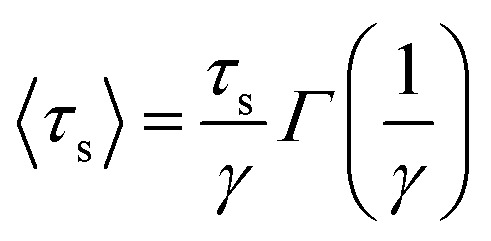
where, *Γ* is the gamma function.

**Table 1:** Residence time of interfacial and bulk water calculated by fitting the survival probability

Cluster	*a*	〈*τ*_s_〉	*γ*	*b*	*τ*2	*c*	*τ*_3_	*n*_p_
NC no = 4 (3.5 Å)	14.2502	17.3738	1.42502	5.36237	3.98729	52.1834	8.89985	0.00068
NC no = 9 (3.5 Å)	23.2337	20.3232	2.32337	17.5998	6.15648	58.7136	10.3767	0.00044
NC no = 14 (3.5 Å)	19.5257	27.0507	1.95273	20.9493	5.94705	99.1797	12.1214	0.00333
NC no = 9 (bulk)	40.0821	25.9479	4.00821	6.59938	−0.417845	65.3204	12.0326	0.01359

From Table 1, it can be observed that different cluster sizes exhibited different *γ* values. This is expected in light of the differences in the cluster sizes and molecule behavior. Table 1 shows that the system with a large number of niclosamide monomers exhibited the slowest dynamics of water, whilst for a system with few niclosamide monomers it exhibited the fastest dynamics. The general trend is that, as the cluster size increases, the ability to trap water molecules inside the shell increases. The bulk water exhibits a different dynamical behavior as compared to the interfacial water molecules near the cluster.

## 4 Conclusions

How water mediates the interaction/assembly of a biological system is an intriguing question that both experimentalists and computational theorists have yet to address precisely. To address this question at the molecular level, we have simulated niclosamide aggregation in water, maybe for the first time. Niclosamide tends to form a large cluster in an orderly manner. Several intermediate metastable clusters are formed during the development of stable large clusters. The dynamical transitions from segregation to aggregation are also monitored. We have also studied the dynamics of water hydration at the interface of the clusters. We showed that water molecules at the interface present rather different dynamical properties compared to the bulk water. The clusters formed have a defined crystalline form. Although this work has described the hydrophobic aggregation of niclosamide in water, problems relating to heterogeneous crystal water interaction, polymorphism growth, solubility measurements, and how they can be addressed by quantitative approaches like the potential mean of force for large hydrophobic clusters are worth pursuing in future.

## Author contributions

All authors conceived and designed the study, DMS and LWK performed MD and DFT experiments and DMS drafted the manuscript. All authors read, revised and approved for submission.

## Conflicts of interest

There are no conflicts to declare.

## Supplementary Material

RA-011-D1RA05358B-s001
