# Supplementary material for: Cation–π interactions drive hydrophobic self-assembly and aggregation of niclosamide in water
Source: RSC Adv. 2021 Oct 7;11(52):33136–47. doi: 10.1039/d1ra05358b (PMC9042188; doi:10.1039/d1ra05358b)
Supplement: RA-011-D1RA05358B-s001 [file RA-011-D1RA05358B-s001.pdf]

Electronic Supplementary Material (ESI) for RSC Advances.
This journal is © The Royal Society of Chemistry 2021

# Electronic Supporting Information (ESI): Cation−π interactions drive hydrophobic self-assembly and aggregation of niclosamide in water

Said A.H. Vuai[1], Mtabazi G. Sahini[1], Isaac Onoka[1], Lucy W. Kiruri[2] Daniel M. Shadrack[3*]

[1]Department of Chemistry, College of Natural and Mathematical Sciences, University of Dodoma, P.O. Box 338 Dodoma, Tanzania.
[2]Department of Chemistry, P.O. Box 43844-00100, Kenyatta University, Nairobi, Kenya
[3] Department of Chemistry, Faculty of Natural and Applied Sciences, St. John's University of Tanzania, P.O. Box 47 Dodoma, Tanzania

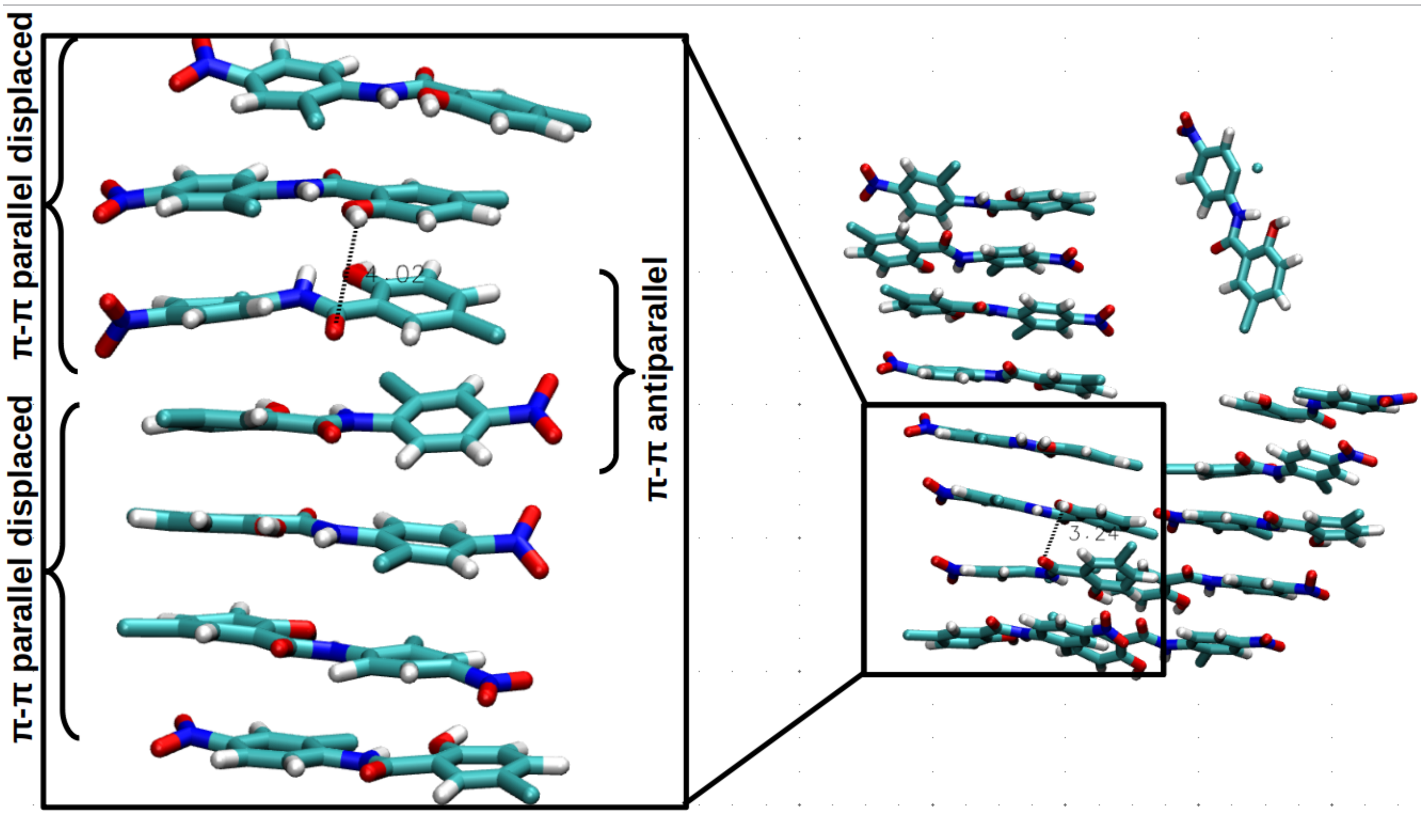


Figure S1: Dominance formation of meta-stable parallel $\pi - \pi$ stacking conformation of niclosamide aggregate for 14 monomers.

*Correspondence: dmshadrack@gmail.com or mshadrack@sjut.ac.tz (DMS)

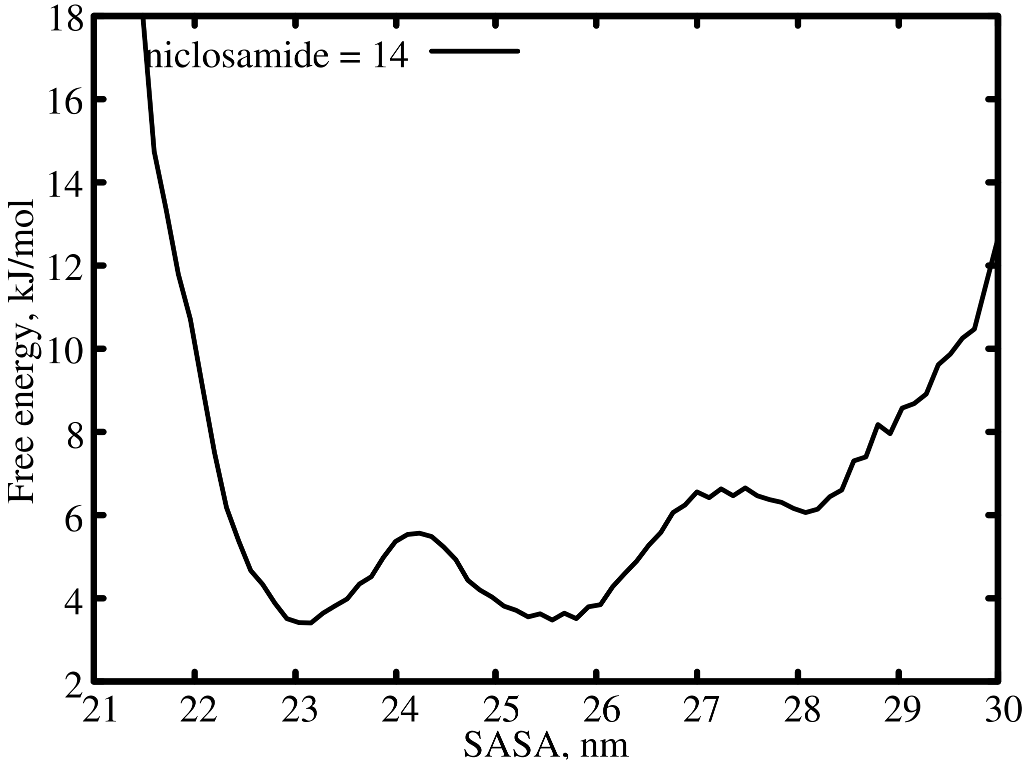


Figure S2: 1D FES for SASA for niclosamide aggregated cluster with 14 monomers.

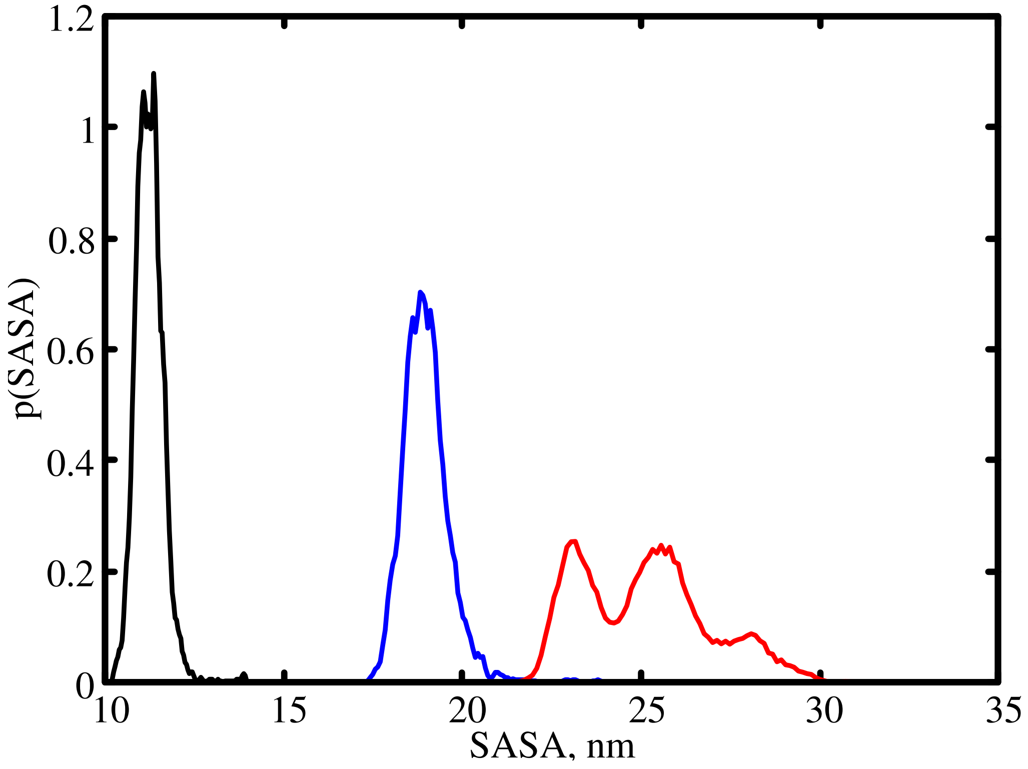


Figure S3: Probability distribution profiles for SASA values. Niclosamide = 4 (black), niclosamide = 9 (blue), and niclosamide = 14 (red).

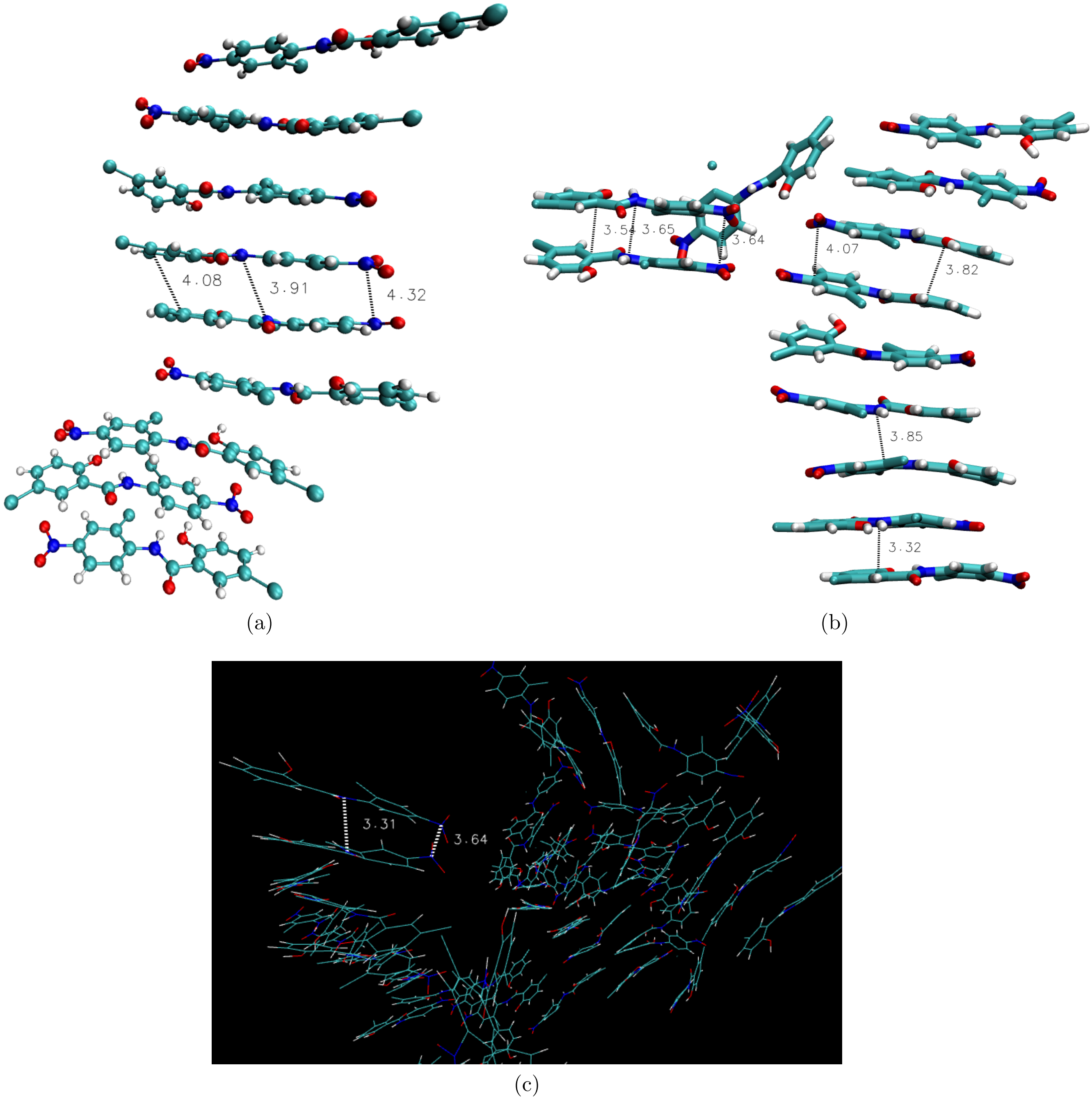


Figure S4: Decreases in distance between parallel fragments for (a) 9 monomers and (b) 14 monomers, for the system with 14 monomers some are not shown. (c) 49 monomers
